# Identification of protein interfaces within the multi‐aminoacyl‐tRNA synthetase complex: the case of lysyl‐tRNA synthetase and the scaffold protein p38

**DOI:** 10.1002/2211-5463.12074

**Published:** 2016-05-25

**Authors:** Azaria Rémion, Fawzi Khoder‐Agha, David Cornu, Manuela Argentini, Virginie Redeker, Marc Mirande

**Affiliations:** ^1^ Laboratoire d'Enzymologie et Biochimie Structurales (LEBS)CNRSGif‐sur‐YvetteFrance; ^2^ Institute for Integrative Biology of the Cell (I2BC)CEACNRSUniv. Paris‐Sud, Université Paris‐SaclayGif‐sur‐YvetteFrance; ^3^ Service d'identification et de Caractérisation des Protéines par Spectrométrie de Masse (SICaPS)CEACNRSUniv. Paris‐Sud, Université Paris‐SaclayGif‐sur‐YvetteFrance; ^4^ Present address: Paris‐Saclay Institute of Neuroscience (Neuro‐PSI)CNRS1 avenue de la Terrasse91190Gif‐sur‐YvetteFrance

**Keywords:** cross‐link, lysyl‐tRNA synthetase, multisynthetase complex, p38, protein:protein interaction

## Abstract

Human cytoplasmic lysyl‐tRNA synthetase (LysRS) is associated within a multi‐aminoacyl‐tRNA synthetase complex (MSC). Within this complex, the p38 component is the scaffold protein that binds the catalytic domain of LysRS via its N‐terminal region. In addition to its translational function when associated to the MSC, LysRS is also recruited in nontranslational roles after dissociation from the MSC. The balance between its MSC‐associated and MSC‐dissociated states is essential to regulate the functions of LysRS in cellular homeostasis. With the aim of understanding the rules that govern association of LysRS in the MSC, we analyzed the protein interfaces between LysRS and the full‐length version of p38, the scaffold protein of the MSC. In a previous study, the cocrystal structure of LysRS with a N‐terminal peptide of p38 was reported [Ofir‐Birin Y *et al*. (2013) *Mol Cell* 49, 30–42]. In order to identify amino acid residues involved in interaction of the two proteins, the non‐natural, photo‐cross‐linkable amino acid *p*‐benzoyl‐l‐phenylalanine (Bpa) was incorporated at 27 discrete positions within the catalytic domain of LysRS. Among the 27 distinct LysRS mutants, only those with Bpa inserted in place of Lys356 or His364 were cross‐linked with p38. Using mass spectrometry, we unambiguously identified the protein interface of the cross‐linked complex and showed that Lys356 and His364 of LysRS interact with the peptide from Pro8 to Arg26 in native p38, in agreement with the published cocrystal structure. This interface, which in LysRS is located on the opposite side of the dimer to the site of interaction with its tRNA substrate, defines the core region of the MSC. The residues identified herein in human LysRS are not conserved in yeast LysRS, an enzyme that does not associate within the MSC, and contrast with the residues proposed to be essential for LysRS:p38 association in the earlier work.

**Abbreviations**
Bpa: *p*‐benzoyl‐l‐phenylalanine
LysRS: lysyl‐tRNA synthetase
MSC: multi‐aminoacyl‐tRNA synthetase complex

The multi‐aminoacyl‐tRNA synthetase complex (MSC) consists of nine aminoacyl‐tRNA synthetases specific for esterification of tRNA with Arg, Asp, Gln, Glu, Ile, Leu, Lys, Met, and Pro, and three auxiliary proteins named p18, p38, and p43 1, 2, 3. The p43 protein associates with ArgRS and GlnRS to build subcomplex 2, p18 associates with MetRS within subcomplex 1, and p38 is the scaffold protein for the assembly of the MSC 4, 5.

This multienzyme complex is thought to be important for integration of protein synthesis in the cytoplasm of eukaryotic cells 6, 7, but it also could be an essential feature of cellular homeostasis through its ability to regulate in space and in time the functioning of several of its components 8. Indeed, specific and controlled release of some of its components was shown to be responsible for essential regulatory functions 9, such as gene‐specific silencing of translation in the case of glutamyl‐prolyl‐tRNA synthetase 10, or by regulating gene expression at the transcriptional level in the case of lysyl‐tRNA synthetase (LysRS) 11. In both cases, phosphorylation of the polypeptide components of the MSC is essential to trigger their dissociation from other components of the complex 12, 13. Following stimulation of cells with IFN‐γ, the WHEP domain in glutamyl‐prolyl‐tRNA synthetase is phosphorylated, which causes its release from the MSC and its association within IFN‐γ activated inhibitor of translation complex, resulting in translational silencing of IFN‐γ activated genes 10. Following stimulation of mast cells, phosphorylation of LysRS on Ser207 induces conformational changes which prevent its association within the MSC, provoke its nuclear translocation, and trigger AP_4_A synthesis and activation of the transcription factor MITF 14. To understand the switch between the translational function of the aminoacyl‐tRNA synthetases associated within the MSC and their mobilization to fulfill other, noncanonical functions, a better knowledge of their structural organization within the MSC is required. Low‐resolution structures of the MSC are available 15, 16 but they are not sufficient to describe, at the atomic level, the protein‐binding sites involved in protein:protein interactions within the MSC.

LysRS is one of the components of the MSC for which most data are available to describe its association in the complex and its release to participate in other cellular functions. LysRS is a dimer that interacts with p38, the scaffold protein of the MSC. This association is mediated by interaction between the catalytic domain of LysRS and the N‐terminal region of p38 4. Although these two proteins form a stable complex *in vitro*, with a dissociation constant of 0.3 nm 17, LysRS is selectively dissociated from the MSC by chaotropic salts 18. Several noncanonical functions were ascribed to LysRS 19. LysRS has long been known to produce Ap_4_A 20, 21, which has been recently shown to regulate the activity of the transcription factor MITF 13. LysRS has also been reported to have the ability to interact with several proteins: with syntenin‐1 which modulates the activity of LysRS 22, and with laminin receptor to control the stability of the receptor 23. Association of LysRS within the MSC, or its recruitment to fulfill noncanonical functions after association with other proteins, involves its alternative and regulated interaction with several proteins. Characterization of protein interfaces involved in the many facets of LysRS function would help to clarify our understanding of these cellular mechanisms.

Our knowledge of protein interfaces between LysRS and its partners is quite limited. The only data concern the cocrystal structure of LysRS in complex with a N‐terminal peptide of p38, the scaffold protein of the MSC 14. To identify the protein–protein interaction sites between LysRS and the native full‐length p38 protein of the MSC, we used a photochemical cross‐linking strategy. By mutagenesis, we incorporated *p*‐benzoyl‐l‐phenylalanine (Bpa) 24, a photoreactive amino acid, at 27 specific positions of the catalytic domain of LysRS in 27 distinct mutants. By characterization of the LysRS^Bpa^ mutants that could be cross‐linked to native full‐length p38, we identified the area of LysRS effectively interacting with p38. Using a targeted mass spectrometric analysis of the two photo‐cross‐linked LysRS^Bpa^–p38 complexes obtained, we identified the p38 region cross‐linked to LysRS. We showed that Bpa inserted at positions 356 or 364 of LysRS interacts with the p38 peptides spanning from Val18 to Arg26 and Pro8 to Arg17, respectively. Our data, obtained with a native full‐length scaffold p38 protein, essentially reveal the same binding area as previously observed with a N‐terminal peptide of p38 14, but suggest that an unstructured peptide does not completely recapitulate the binding site of LysRS on p38.

## Results

### Expression of LysRS mutants containing Bpa

**Figure 1:**
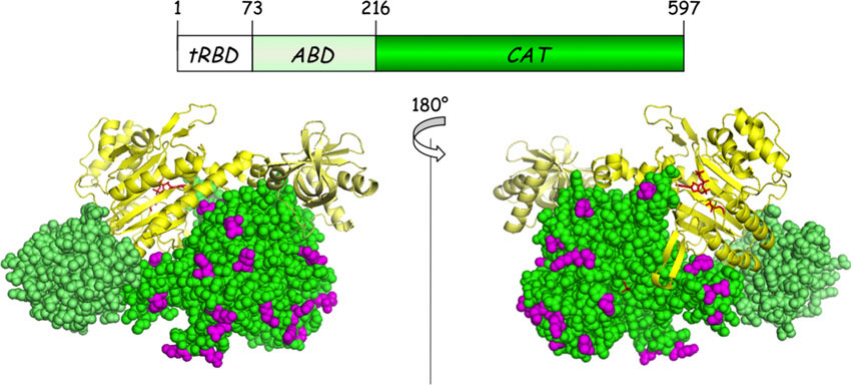
Localization of amino acid residues mutated into Bpa. (Top) Schematic representation of a subunit of human cytoplasmic LysRS composed of a eukaryote‐specific N‐terminal tRNA‐binding domain (tRBD), an anticodon‐binding domain (ABD), and a catalytic domain (CAT). (Bottom) 3D representation of a dimer of LysRS crystallized in the absence of its N‐terminal tRBD (3BJU). One monomer is in yellow, the other in green. ABDs are in light color. Side chains of amino acid residues that were mutated into Bpa are shown in purple.

**Figure 2:**
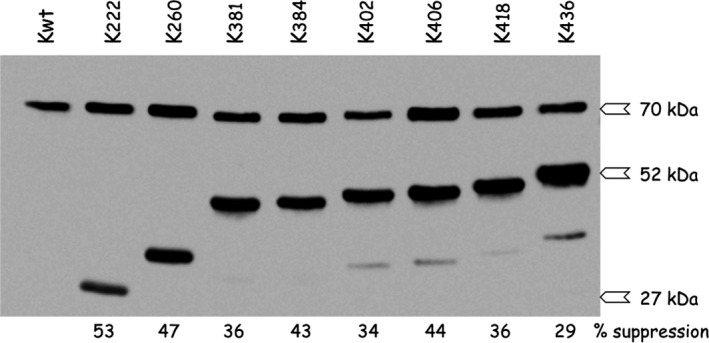
Expression of a subset of Kxxx mutants and efficiency of amber codons suppression. The efficiency of suppression in *E. coli* cells expressing the orthogonal Bpa aminoacylation system was monitored by western blotting with polyclonal antibodies directed to human LysRS. An amber stop codon was incorporated at position xxx of cDNA encoding LysRS, and expression in the presence of Bpa led to the synthesis of a full‐length protein of 70 474 Da when Bpa was inserted at the positions of stop codons. Suppression efficiencies (in %) are indicated below the blot. When suppression was not complete, a truncated LysRS product was synthesized from ATG1 to residue xxx (Table 1), ranging from 27 191 Da (K222) to 52 242 Da (K436).

We previously determined that LysRS directly associates with p38, the scaffold protein of the MSC 4. This association is robust (*K*_d_ of 0.3 nm) 17 and involves the catalytic domain of LysRS, from residues 216 to the C terminus 25. Based on the 2.3 Å crystal structure of a fragment of human LysRS containing the catalytic domain 26, the side chains with the highest score of accessible area were selected using the ccp4 suite software 27. Among them, 27 positions (Table 1), corresponding to amino acid side chains evenly distributed at the surface of the catalytic domain, were selected for insertion of the non‐natural genetically encoded photoactivable and cross‐linkable amino acid Bpa (Fig. 1). LysRS was expressed in *E. coli* BL21(DE3) that also contains pEVOL‐Bpa expressing the orthogonal suppression system, namely *Mj*tRNA^Tyr,CUA^, an amber suppressor tRNA derived from *M. jannaschii* tRNA^Tyr^, and *Mj*TyrRS^Bpa^ a mutant of *M. jannaschii* tyrosyl‐tRNA synthetase that specifically aminoacylates *Mj*tRNA^Tyr,CUA^ with Bpa 24. In the absence of Bpa in the culture medium, no full‐length LysRS was synthesized (result not shown). After addition of Bpa, suppression was not complete, but its efficiency ranged from 25% to 60% according to the position of the amber codon in mRNA carrying the mutation (see Fig. 2 for a selection of mutants). The 27 mutants of LysRS (named Kxxx) carrying a single Bpa inserted at the position indicated [Kxxx indicates the position xxx in the amino acid sequence of LysRS (K)] were purified as described in Materials and methods. During purification of the various mutants, LysRS^Bpa^ were eluted from the Mono S column as fairly symmetrical peaks, which suggested that insertion of Bpa did not impair protein folding. Crystallographic studies on the complex between gankrin and S6 proteosomal protein also showed that cross‐linked structure exhibits little structural distortion from the native complex, which validated the choice of Bpa incorporation to probe protein–protein interactions 28.

**Table 1:** Position of Bpa insertion into LysRS

Residue	Position in LysRS^a^	*M*_r_ (kDa)^b^
Asp	222	27.191
Phe	239	29.384
Glu	260	32.010
Glu	267	32.812
Asp	291	35.438
Lys	356	43.201
His	364	44.062
Lys	370	44.721
Glu	379	45.716
Gln	381	45.902
Asp	384	46.265
Arg	392	47.184
Glu	398	47.953
Lys	402	48.454
Met	406	48.823
Glu	410	49.293
Glu	418	50.257
Lys	421	50.643
Val	428	51.444
Pro	436	52.242
Arg	477	56.914
Gln	510	60.930
Lys	517	61.775
Ala	520	62.103
Glu	531	63.239
Phe	570	67.509
Glu	576	68.181
WT		70.474

a Numbering is according to PDB file 3BJU.

b Expected *M*_r_ when expressed in pET28b in the absence of suppression.

### Identification of the amino acid residues of LysRS involved in its interaction with p38, the scaffold protein of the MSC

**Figure 3:**
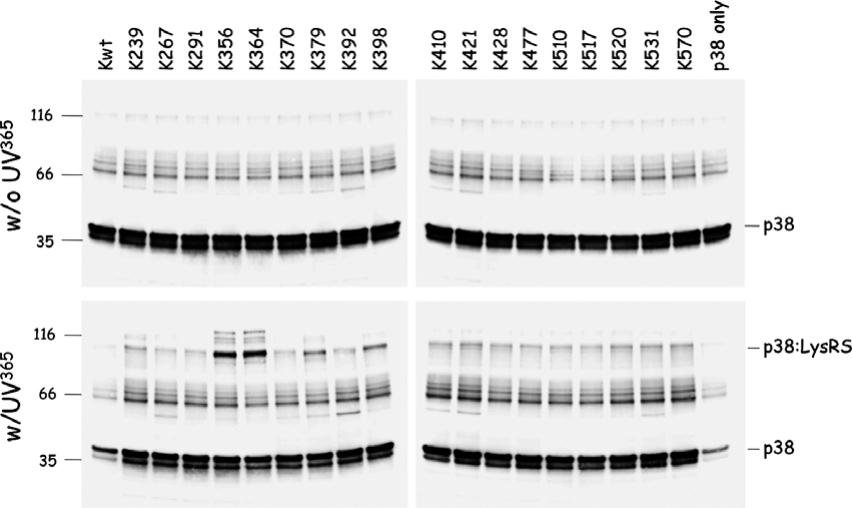
Cross‐linking of Kxxx variants with p38. Wild‐type LysRS (Kwt) or LysRS species carrying a Bpa at position xxx (only a subset of Kxxx variants are shown) were incubated at a dimer concentration of 70 nm in the presence of p38 (350 nm), during 60 min, with (w/UV ^365^) or without (w/o UV ^365^) exposure to UV light at 365 nm. After incubation, samples were denatured in sample buffer containing SDS, subjected to SDS/PAGE and western blotting with antibodies directed to p38. Size markers are indicated on the left (in kDa), and p38 polypeptide or p38 cross‐linked with LysRS (p38:LysRS) are identified on the right.

To determine the surface area of LysRS involved in its interaction with p38, the scaffold protein of the MSC 4, each of the 27 LysRS mutants (Kxxx) containing a single Bpa per polypeptide chain, and wild‐type LysRS that does not contain Bpa (Kwt), were incubated in the presence of purified p38. After 60 min of exposure to UV at 365 nm, the samples were analyzed by SDS/PAGE and western blotting using anti‐p38 antibodies (Fig. 3). Among the 27 incubations with Kxxx variants, two of them, K356 and K364, corresponding to Bpa inserted at positions 356 or 364 of LysRS, showed the presence of an abundant high‐molecular‐mass product of about 100 kDa corresponding to the expected size for a cross‐linked species containing one molecule of p38 per molecule of LysRS. This cross‐linked polypeptide was not observed in the absence of exposure to UV light at 365 nm, and was recognized by antibodies directed to p38 (Fig. 3) and to LysRS (not shown). Analysis by LC‐MS/MS performed after in‐gel enzymatic digestion of these two cross‐linked LysRS^Bpa^–p38 complexes confirmed the presence of both LysRS and p38 in these complexes (see below).

**Figure 4:**
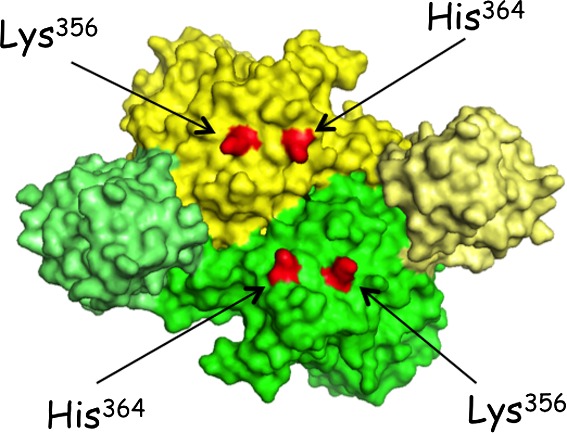
Identification of Lys^356^ and His^364^ in the 3D structure of dimeric LysRS. A dimer of LysRS is represented, with one monomer in yellow, the other in green. ABDs are in light color. Side chains of residues Lys^356^ and His^364^ are shown in red on the two monomers.

In wild‐type LysRS, the two residues at positions 356 and 364 are a Lys and a His residue, respectively. They are distant of only 15 Å on the catalytic domain of one monomer, and the two corresponding residues from the other monomer are nearly 30 Å away, on the same side of the dimer (Fig. 4). They are located on the side of the dimer that is opposite to the site of interaction with the two tRNA molecules. These four residues identify on LysRS the surface area for interaction with p38 (Fig. 4).

### Identification of the peptides of p38 interacting with the LysRS^Bpa^ variants K356 and K364

**Figure 5:**
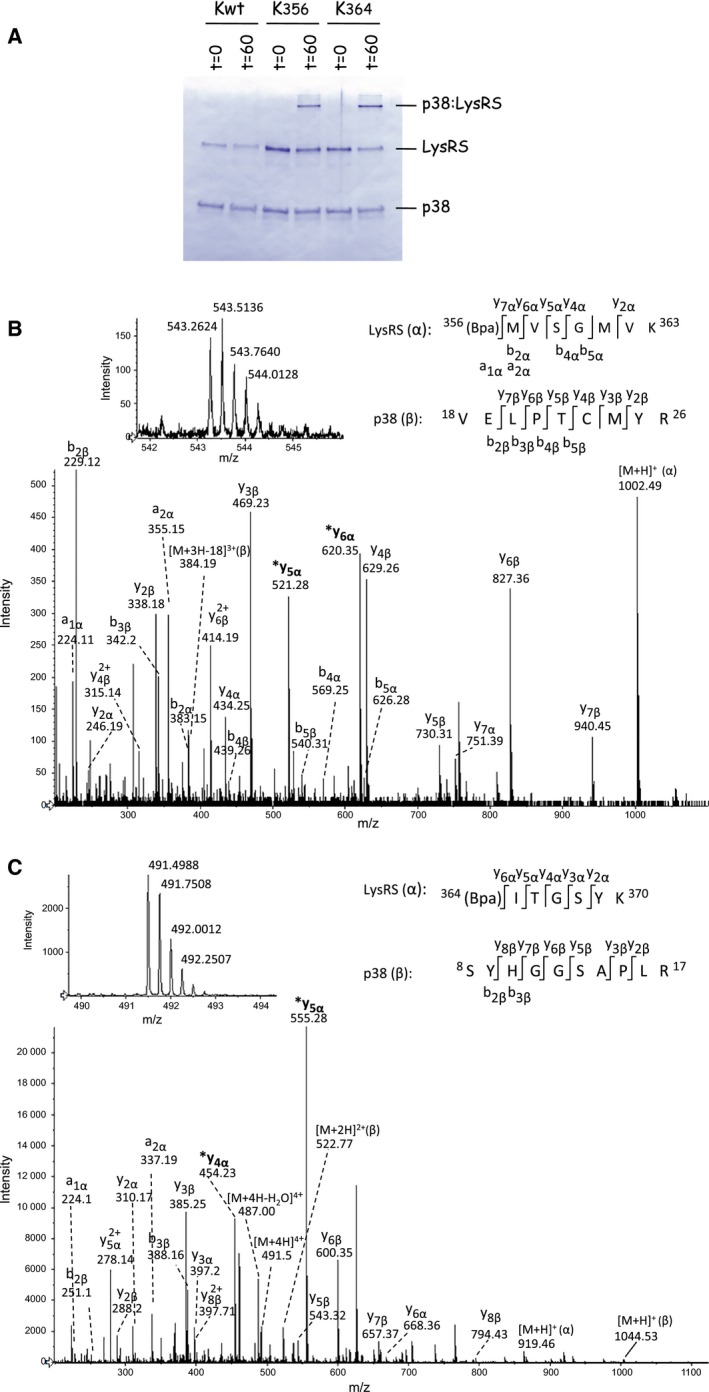
Identification of the p38 peptides cross‐linked to Bpa inserted at positions 356 and 364 of LysRS. (A) Analysis by SDS/PAGE and Coomassie staining of complexes formed after cross‐linking of LysRS and p38. Wild‐type LysRS (Kwt) or LysRS with Bpa inserted at positions 356 (K356) or 364 (K364) were incubated with p38 as described in the legend of Fig. 3. Cross‐linked complexes (p38:LysRS) formed after 60 min of UV exposure were in‐gel digested and the proteolytic peptides were analyzed by LC‐MS/MS analysis. (B) Identification of the p38 peptide cross‐linked to peptide Bpa356‐Lys363 from LysRS with Bpa incorporated at position 356. Upper left panel shows the mass spectrum of the quadruply charged cross‐linked peptide with a monoisotopic *m*/*z* 543.2624 (mass accuracy Δm = 6 p.p.m.). Lower panel corresponds to the fragmentation spectrum of the precursor ion at *m*/*z* 543.2624. Identified fragments and their charge states are indicated. The identified fragments are indicated on the cross‐linked sequences in the upper right panel. The α‐ and β‐sequences correspond to the Bpa356‐Lys363 and the Val18‐Arg26 peptides of LysRS and p38, respectively. Peaks labeled with an asterisk indicate fragments (y_5α_ and y_6α_) used for the targeted cross‐linked peptide search in the LC‐MS/MS dataset. (C) Identification of the p38 peptide cross‐linked to peptide Bpa364‐Lys370 from LysRS with Bpa incorporated at position 364. Upper left panel shows the mass spectrum of the quadruply charged cross‐linked peptide with a monoisotopic *m*/*z* 491.4988 (mass accuracy Δm = 16 p.p.m.). Lower panel corresponds to the fragmentation spectrum of the precursor ion at *m*/*z* 491.4988. Identified fragments and their charge states are indicated. The identified fragments are indicated on the cross‐linked sequences in the upper right panel. The α‐ and β‐sequences correspond to the Bpa364‐Lys370 and the Ser8‐Arg17 peptides of LysRS and p38, respectively. Peaks labeled with an asterisk indicate fragments (y_4α_ and y_5α_) used for the targeted cross‐linked peptide search in the LC‐MS/MS dataset.

To identify the peptides of p38 which were cross‐linked to the Bpa residues inserted within the catalytic domain of LysRS, the 100 kDa high‐molecular‐mass polypeptides observed after electrophoresis on SDS/polyacrylamide gel following exposure to UV (Fig. 5A) were excised from the gel and subjected to trypsin digestion, or to a double digestion with trypsin and Glu‐C proteases, for peptides cross‐linked with Bpa inserted at position 364 or 356 of LysRS, respectively. The proteolytic peptides were characterized by LC‐MS/MS analyses. LysRS and p38 sequence coverage was 70% and 85% for the K364–p38 cross‐linked complex, respectively, and 74% and 78% for the K356–p38 cross‐linked complex, respectively. Although the stavrox software unambiguously identified the K364–p38 cross‐linked peptide generated by trypsin digestion of the protein complex, we had to optimize a targeted search for the identification of the Bpa‐containing cross‐linked peptide generated by the double proteolytic digestion of K356–p38 protein complex. The targeted manual search of diagnostic fragment ions specific of the Bpa‐containing proteolytic peptides of LysRS, performed as described in Materials and methods, successfully detected the LysRS^Bpa^–p38 cross‐linked peptides in the nanoLC‐MS/MS dataset for both the LysRS^Bpa^ variants. The sequence of the p38 peptides cross‐linked to either Bpa356‐Lys363 or Bpa364‐Lys370 peptides of LysRS^Bpa^ was further identified by analysis of the fragmentation mass spectra of the cross‐linked peptides (Fig. 5B,C). The two fragmentation mass spectra (Fig. 5) unambiguously identified that peptides Bpa356‐Lys363 and Bpa364‐Lys370 of LysRS were cross‐linked to peptides Val18‐Arg26 and Ser8‐Arg17 of p38, respectively (Fig. 5B,C). Further attempts to define the exact position of the cross‐link site within the p38 sequences were unsuccessful because the Bpa cross‐link appeared to be labile during MS/MS analysis and exhibited a tendency to fragment easily. Collectively, the amino‐terminal amino acid residues of p38, from residues Ser8 to Arg26, build the binding site of LysRS on the scaffold protein of the MSC.

## Discussion

**Figure 6:**
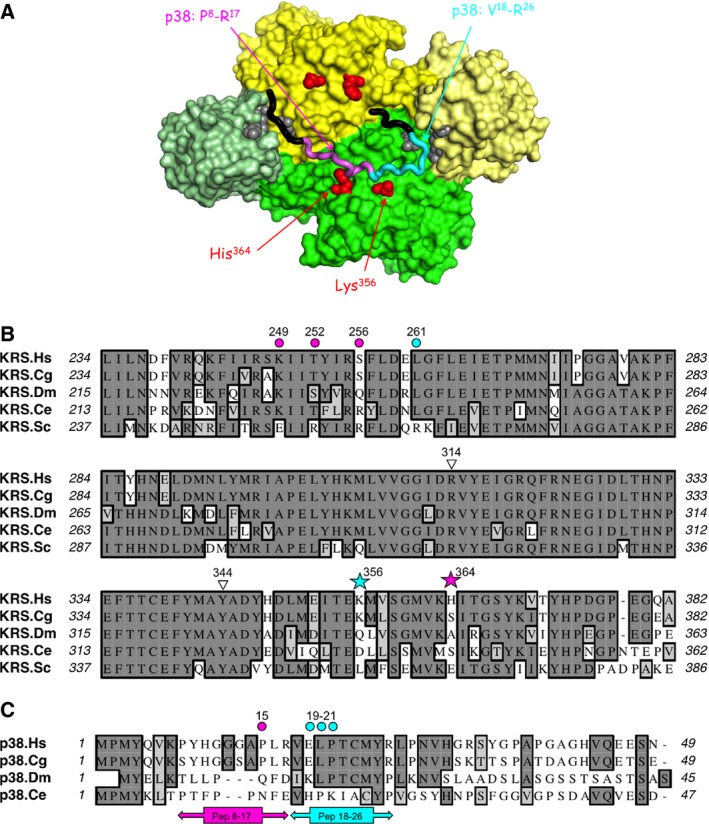
Analysis of the LysRS:p38 interaction site. (A) Identification in the cocrystal structure of ∆N‐LysRS with a N‐terminal peptide of p38 (4DPG), of p38 peptides cross‐linked with Bpa inserted at positions 356 and 364 of LysRS. The dimer of LysRS is represented as in Fig. 4, with residues Lys356 and His364 shown in red. The p38 peptide from Pro2 to His31 is shown, in magenta (from Pro8 to Arg17), cyan (from Val18 to Arg26), and black (from Pro2 to Lys7 and from Leu27 to His31). The peptide in cyan was cross‐linked to Lys356, in magenta to His364. Residues Val101, Ile103, Arg314, and Tyr344 in ∆N‐LysRS are shown in gray. (B) Alignment of the amino acid sequence of LysRS from *Homo sapiens* (KRS.Hs) from residues 234–382 with the corresponding sequences of LysRS from *Cricetilus griseus* (Cg), *Drosophila melanogaster* (Dm), *Caenorhabditis elegans* (Ce), and *Saccharomyces cerevisiae* (Sc). Residues Lys^356^ and His^364^ are indicated by stars. Other residues located less than 4 Å away from p38 are indicated by circles. Residues Arg^314^ and Tyr^344^ are indicated by triangles. (C) Alignment of the amino acid sequence of p38 from *Homo sapiens* (p38.Hs) from residues 1–49 with the corresponding sequences of p38 from *Cricetilus griseus* (Cg), *Drosophila melanogaster* (Dm), and *Caenorhabditis elegans* (Ce). Residues located less than 4 Å away from LysRS are indicated by circles. Residues from LysRS interacting with residues from peptide 8–17 of p38, or interacting with residues from peptide 18–26 of p38, are indicated in magenta or cyan, respectively.

We previously established that p38 forms a tight complex with LysRS (*K*_d_ of 0.3 nm) 17. Association of LysRS to p38 only involves the catalytic domain of LysRS; no interaction was detected between p38 and the anticodon‐binding domain of the synthetase or with its eukaryote‐specific N‐terminal appended domain 25. The very N‐terminal domain of p38, from residues 1 to 42, is sufficient to associate with LysRS 4. In the present study, using site‐directed insertion of the photo‐cross‐linkable Bpa at specific positions of the catalytic domain of LysRS, we located the regions of LysRS involved in the interaction with full‐length p38 at position 356 and 364, and we identified that the p38 peptide spanning from position 8 to 26 interact with the two LysRS positions of the interface between LysRS and p38. The reactive volume of benzophenones corresponds to a sphere with a radius of about 3–4 Å from the ketone oxygen 29. Taking into account the cocrystal structure of ∆N‐LysRS, a LysRS derivative with a deletion of the 70 N‐terminal residues, with a N‐terminal 48 amino acid residue peptide of p38 14, we observed that Lys356 of LysRS is located less than 4 Å away from residues Glu19, Leu20, and Pro21 from peptide Val18‐Arg26 of p38, and that residue His364 of LysRS is located less than 4 Å away from residue Pro15 from peptide Ser8‐Arg17 of p38 (Fig. 6A,C). The cocrystal structure also suggests that residues Lys249, Thr 252, Ser256, or Leu261 of LysRS are located less than 4 Å away from peptides Ser8‐Arg17 or Val18‐Arg26 of p38, at a distance compatible to form the LysRS:p38 binding site (Fig. 6A,B). It is noteworthy that among these six residues of the catalytic domain of LysRS (Lys249, Thr252, Ser256, Leu261, Lys356, and His364), which could form the binding site of p38, none is conserved in yeast LysRS (Fig. 6B), an enzyme that cannot associate with p38 in cellulo 30.

In the cocrystal structure of ∆N‐LysRS with a N‐terminal 48‐amino acid residue peptide of p38 14, the peptide regions 3–6 and 24–27 of p38 were observed to interact with residues Val101 and Ile103 from one monomer of LysRS and with Arg314 and Tyr344 from the other monomer (Fig. 6A). Taking into account that residues Val101 and Ile103 are located within the tRNA anticodon‐binding domain of LysRS that does not interact with p38 25, and that the four LysRS residues Val101, Ile103, Arg314, and Tyr344 are conserved in yeast LysRS (Fig. 6B), an enzyme that does not associate with p38 30, we suggest that the N‐ and C‐terminal extremities of the peptide used in that study 14 do not completely recapitulate the folding of these two regions within the native, full‐length p38 used in our study. In the crystal structure of ∆N‐LysRS with a N‐terminal peptide of p38, it is probable that the peptide does not fold into the native conformation that it adopts in the full‐length p38 protein, thus leading to unstructured peptide segments on both sides of the specific contact areas, that either were not visible in the crystal, as reported for residues 32–48 14, or that made nonspecific contacts with LysRS, as suggested here for residues Met3‐Tyr‐Gln‐Val6 and Met24‐Tyr‐Arg‐Leu27, which are especially hydrophobic and were observed lying in a hydrophobic groove formed at the interface between the two subunits of dimeric LysRS (Fig. 6A). The cocrystal structure of LysRS with native p38 should help to clarify this issue. However, it is striking to observe that independent approaches performed with purified full‐length p38 and LysRS proteins (this study) or with purified ∆N‐LysRS and a 48‐amino acid residue N‐terminal peptide of p38 14 led to the identification of a similar region in LysRS that interacts with a folded p38 protein or with an unfolded peptide. This suggests that the p38–peptide 1–48 used in the cocrystallization approach 14 is capable of adopting in part a native conformation upon interaction with LysRS. Previous analyses revealed that association of LysRS with p38 is realized through a very slow association rate constant (*k*_on_ of 90 × 10^3^ m ^−1^·s^−1^), also combined with a slow dissociation rate (*k*_off_ of 30 × 10^−6^ s^−1^) leading to an equilibrium dissociation constant *K*_d_ of 0.3 nm 17. Such a slow *k*_on_ is unusual for protein:protein interactions, and suggests that even within the full‐length folded p38, the region of association with LysRS should undergo discrete conformational changes for association to occur.

The p38 protein, the scaffold protein of the MSC, was shown to bind directly several components of the complex, including p43, AspRS, ArgRS, LysRS, GlnRS, and GluProRS 4. Dimeric LysRS was found to form the most stable association with p38, with a *K*_d_ value of 0.3 nm 17. Because the MSC may accommodate one or two dimers of LysRS, and one dimer of p38 per molecule of complex 17, 31, it is possible that each monomer of p38 may bind one LysRS dimer 14, 32 or that one dimer of p38 associates one dimer of LysRS per molecule of MSC. The p38 interaction sites on each monomer of LysRS are localized on the same face of the dimer and are at a distance of about 30 Å (Fig. 6A), which is consistent with one dimer of p38 binding to one dimer of LysRS. Interestingly, MAPK‐dependent phosphorylation of LysRS on Ser207 is necessary to operate the switch between its translational function, when associated within the MSC in the cytoplasm, and its nuclear function in the regulation of the transcription factor MITF 13, 14. Phosphorylation on Ser207 induces a large conformational change on LysRS, with the tRNA anticodon‐binding N‐terminal domain of LysRS moving 15 Å away from the catalytic domain 14. This results in the inactivation of LysRS in the tRNA aminoacylation reaction and prevents its association with p38 14. While the outer surface of LysRS is exposed to the solvent and capable of interacting with tRNA, its inner surface, defined by the p38‐interacting area, should not be easily accessible to a kinase due to its interaction with p38 and association with the other components of MSC. Thus, it is probable that phosphorylation targets newly synthesized molecules of LysRS, before their association within the MSC, rather than molecules of LysRS already assembled. Transcription factors are among the less abundant proteins in the nucleus, and only few molecules of LysRS are certainly involved in this regulatory pathway.

Among components of the MSC, some aminoacyl‐tRNA synthetases have acquired in evolution a specific domain involved in protein:protein interactions within the complex. It is the case of methionyl‐tRNA synthetase or arginyl‐tRNA synthetase, for which a eukaryote‐specific N‐terminal domain is required for their association within the MSC via interaction with the p18 or p43 components, respectively 33, 34. By contrast, concerning glutaminyl‐ or lysyl‐tRNA synthetase, the core catalytic domain of the synthetases is involved in their association to components of the MSC 25, 35. Because the homologous enzymes from bacteria or yeast do not associate within complexes, this suggests that their catalytic domains have evolved new protein:protein interfaces which allowed human enzymes to associate with other components. The protein interfaces identified in this study for association of LysRS with p38 are not totally conserved in evolution even for pairs of proteins that do associate within a MSC in more distantly related species such as in the fly *D. melanogaster* 36 or in the worm *C. elegans* 37 (Fig. 6B,C). Some of the residues identified in this study are conserved in human, hamster, fly, and worm, but some of them are divergent in the fly or the worm (Fig. 6). As a general rule, divergence of residues in LysRS is always associated with a divergence in the N‐terminal peptide of p38, consistent with coevolution of the two complementary protein areas to maintain a stable dimeric interface. Adaptation in evolution of the accessible surface areas of LysRS and p38 was certainly required to build mutually competent protein:protein interfaces.

## Materials and methods

### Expression of LysRS in *E. coli*

Full‐length human LysRS was expressed in *E. coli* BL21 (DE3) after subcloning into the *Nhe*I and *Xho*I sites of pET‐28b. The N‐terminal sequence MGSSHHHHHHSSGLVPRGSHMAS is encoded by the plasmid.

### Site‐directed mutagenesis

The QuickChange Lightning Site‐directed Mutagenesis Kit from Agilent Technologies was used to introduce amber (TAG) stop codons at discrete sites within the nucleotide sequence encoding the catalytic domain of LysRS. The numbering of amino acid residues refers to their numbering in the full‐length cytoplasmic LysRS for which the 3D structure of a fragment from residue 70–576 has been determined 26. The pET‐28b/Kxxx plasmid expresses a LysRS protein (K) mutant carrying an amber codon at position xxx that will be translated into Bpa using the orthologous suppression system developed by P. Schultz 24. Distinct LysRS variants were produced by insertion of Bpa at the 27 distinct positions listed in Table 1.

### Purification of proteins

Incorporation of Bpa into mutant LysRS proteins was conducted in *E. coli* BL21(DE3) transformed with pET‐28b/Kxxx and pEVOL‐Bpa (gift of P. Schultz), grown at 37 °C in 1L of LB medium supplemented with kanamycin (50 μg·mL^−1^) and chloramphenicol (34 μg·mL^−1^), and containing 0.2% arabinose and 1 mm Bpa (Bachem). When the culture reached an *A*_600_ = 1.0, expression was induced by addition of 1 mm IPTG for 5 h. Cells were washed twice with ice‐cold buffer 150/10 (20 mm K‐phosphate pH 7.5, 150 mm NaCl, 10 mm imidazole, 5% glycerol, 5 mm 2‐mercaptoethanol), resuspended in the same buffer (1 millilitre per gram of cell pellet), and lysed in an Eaton Press after freezing in dry ice. All subsequent steps were conducted at 4 °C. After addition of 1 V of buffer 500/10 (20 mm K‐phosphate pH 7.5, 500 mm NaCl, 10 mm imidazole, 5% glycerol, 5 mm 2‐mercaptoethanol) and of protease inhibitors (1 mm pefabloc, 10 mm benzamidine, and 10 mm PMSF), extracts were clarified by sonication and by centrifugation at 20 000 ***g*** for 20 min, and at 70 000 ***g*** for 1 h. The clear supernatant was incubated with 500 μL of Ni‐NTA Superflow (Qiagen) for 1 h at 4 °C. The matrix was washed twice by centrifugation/resuspension in buffer 500/10, and poured in a Micro BioSpin column (Biorad). Beads were washed with 5 x 1 mL of buffer 500/10, and elution was performed by adding 4 x 1 mL of buffer 500/400 (20 mm K‐phosphate pH 7.5, 500 mm NaCl, 400 mm imidazole, 5% glycerol, 5 mm 2‐mercaptoethanol). After dialysis against buffer A‐S [20 mm Tris/HCl (pH 7.5), 50 mm KCl, 10% glycerol, 10 mm 2‐mercaptoethanol], samples were applied to a Mono S HR 5/5 column equilibrated in the same buffer, and proteins were eluted by a linear gradient (50 column vol.) of KCl from 50 to 350 mm. Fractions containing the Kxxx mutant proteins were concentrated by ultrafiltration (Vivaspin 6, 10 kDa), dialyzed against PBS (136 mm NaCl, 2.7 mm KCl, 8 mm Na_2_HPO_4_, 1.47 mm KH_2_PO_4_), and stored at −80 °C.

The p38 component of the MSC from hamster (*Cricetilus griseus*) was expressed in *E. coli* and purified as described previously 17.

### Protein cross‐linking

Photo‐cross‐linking was conducted essentially as described previously 38. Wild‐type LysRS (Kwt) or LysRS with Bpa inserted at position xxx (Kxxx) and p38 were dialyzed against PBS. The different LysRS species, at a dimer concentration of 70 nm, and p38, at a dimer concentration of 350 nm, were mixed in a final volume of 80 μL into the wells of a 96‐well plate cooled on ice. Plate was covered with its polystyrene lid and with a 3‐mm glass plate to filter short‐wavelength UV light, and incubated into a CL‐1000 Ultraviolet Crosslinker (UVP) equipped with a 365 nm UV lamp. Control samples were withdrawn before starting irradiation, and cross‐linked products were analyzed by SDS/PAGE after a 60‐min exposure to UV light.

### Antibodies and western blot analysis

Polyclonal antibodies to LysRS and p38 have been described previously 39. Western blot analyses were conducted in a BenchPro 4100 station (Life Technologies) with goat anti‐rabbit secondary antibodies conjugated with peroxidase (Chemicon) and the SuperSignal West Pico chemiluminescent substrates (Pierce). Chemiluminescence was detected with a LAS‐3000 Imaging System (Fuji).

### Peptide preparation

Bands corresponding to p38, LysRS, and LysRS–p38 cross‐linked complexes were excised from the gel after SDS/PAGE and Coomassie blue staining. Washing, reduction, and alkylation of excised bands were performed using the Progest robot (Genomic Solutions, Chemsford, MA, USA) as described previously 40. Trypsin digestion was performed overnight at room temperature after addition of 20 μL of 10 ng·μL^−1^ Porcine Gold Trypsin (Promega, Madison, WI, USA) in 25 mm NH_4_HCO_3_. Double sequential digestion with trypsin/Glu‐C was performed by addition of 20 μL of 20 ng·μL^−1^ Glu‐C from *Staphylococcus aureus* V8 (Roche) in 25 mm NH_4_HCO_3_ after tryptic digestion, and an additional overnight digestion at room temperature. Finally, proteolytic peptides were extracted by addition of 20 μL of 60% CH_3_CN and 0.1% formic acid and a 2‐h incubation. Extracted peptides were vacuum dried and resuspended in 0.1% formic acid prior to mass spectrometry analyses.

### NanoLC‐MS/MS analysis

Peptide digests were analyzed by nanoLC‐MS/MS using a nanoRSLC HPLC system (Dionex, Thermo‐Scientific, Waltham, MA, USA) coupled to the nanoelectrospray ion source of a Triple‐TOF 4600 mass spectrometer (ABSciex). Peptides were loaded on a trap column (Acclaim PepMap100C18, 75 μm i.d. × 2 cm, 3 μm) at a flow rate of 5 μL·min^−1^ of loading buffer. The loading buffer was H_2_O/CH_3_CN/TFA (98%/2%/0.05%). Peptide separation was performed on a reverse phase C18 analytical column (Acclaim PepMapRSLCC18, 75 μm i.d. × 15 cm, 2 μm, 100 Å) at a flow rate of 300 nL·min^−1^ using a gradient of 5–35% CH_3_CN for 40 min. Solvent A and B were 0.1% formic acid in water and 0.1% formic acid in CH_3_CN, respectively. MS/MS spectra were acquired with a data‐dependent acquisition method by selecting the 10 most intense precursors for CID fragmentation with Q1 quadrupole set at low resolution for better sensitivity.

### Data processing

NanoLC‐MS/MS raw files were converted to mgf files using ms data converter software before analysis with peakview software (ABSciex). NanoLC‐MS/MS data were processed automatically using the mascot (version 2.4.1) search engine with the following modifications: carbamidomethylation of cysteines as fixed modification, oxidation of methionines as variable modifications, and a mass increment due to substitution of lysine or histidine by Bpa for K356 and K364, respectively. The mass increment was 122.9996 Da and 114.0357 Da for Lys to Bpa replacement in K356 mutant and His to Bpa replacement in K364 mutant, respectively. Peptide and fragment tolerance were respectively set at 20 p.p.m. and 0.05 Da. Only peptides identified with a mascot score higher than 25 were considered. Cross‐linked peptides were searched using the stavrox software (3.4 version) 41 and a targeted analysis of the nanoLC‐MS/MS data was performed manually in order to specifically identify the cross‐linked peptides composed of the Bpa‐containing proteolytic peptides of LysRS. For each LysRS^Bpa^ mutant, the fragmentation mass spectra of the Bpa‐containing proteolytic peptide was analyzed for the noncross‐linked LysRS protein band in order to identify specific fragment ions, and later on used for the targeted identification of cross‐linked LysRS–p38 peptides. This analysis also confirmed that the LysRS variants were fully modified, and contained Bpa inserted at the expected position. For the K356 mutant cross‐linked to p38, the LysRS peptide Bpa356‐Lys363 produced by double trypsin/Glu‐C digestion was detected by the targeted search of fragment ions y6 and y5. For the K364 variant, the LysRS peptide Bpa364‐Lys370 generated by trypsin digestion was detected by the targeted search of fragment ions y5 and y4. Using this targeted search, the LysRS^Bpa^–p38 cross‐linked peptides were unambiguously detected in merged mass spectra, and the identity of the p38 peptides cross‐linked to LysRS was deduced from both the monoisotopic mass of the LysRS^Bpa^–p38 cross‐linked peptide and from the fragment ions of p38 identified in the corresponding fragmentation mass spectra.

## Author contributions

VR and MM planned experiments; AR, FKA, DC, MA, and MM performed experiments; VR and MM analyzed data and wrote the paper.
